# Western Cape Primary Care Assessment Tool (PCAT) study: Measuring primary care organisation and performance in the Western Cape Province, South Africa (2013)

**DOI:** 10.4102/phcfm.v8i1.1057

**Published:** 2016-05-19

**Authors:** Graham Bresick, Abdul-Rauf Sayed, Cynthia le Grange, Susheela Bhagwan, Nayna Manga, Derek Hellenberg

**Affiliations:** 1 Division of Family Medicine, School of Public Health and Family Medicine, Health Sciences Faculty, University of Cape Town, South Africa

## Abstract

**Background:**

Major health sector reform and the need for baseline measures of performance to determine impact.

**Aim:**

Baseline audit of primary healthcare (PHC) performance.

**Setting:**

Cape Town and Cape Winelands (rural) PHC facilities (PCFs) in Western Cape Province, South Africa.

**Method:**

The South African cross-culturally validated ZA PCAT to audit PHC performance on 11 subdomains associated with improved health and reduced costs. Adult PCF users systematically sampled. All full-time doctors and nurse practitioners in PCFs sampled and all PCF managers in sub-districts sampled invited into the study.

**Results:**

Data from 1432 users, 100 clinicians and 64 managers from 13 PCFs in 10 sub-districts analysed (figures show stakeholder percentages scoring subdomain performance ‘acceptable to good’). 11.5% users scored access ‘acceptable to good’; community orientation and comprehensive services provided 20.8% and 39.9%, respectively. Total PHC score for users 50.2%; for managers and practitioners 82.8% and 88.0%, respectively. Among practitioners access was lowest (33.3%); PHC team (98.0%) and comprehensive services available (100.0%) highest. Among managers, access (13.5%) and family centredness (45.6%) are lowest; PHC team (85.9%) and comprehensive services available (90.6%) highest. Managers scored access, family centredness and cultural competence significantly lower than practitioners. Users scored comprehensive services available, comprehensive services provided and community orientation significantly lower than practitioners and managers.

**Conclusion:**

Gaps between users’ experience and providers’ assessments of PHC performance are identified. Features that need strengthening and alignment with best practice, provincial and national, and health policies are highlighted with implications for practitioner and manager training, health policy, and research.

## Introduction

Rising healthcare costs and the quadruple burden of disease demand critical scrutiny of healthcare organisation and practice globally. It is estimated that by 2020 chronic diseases will account for 73% of all deaths and 60% of the global burden of disease.^1^ The growing burden of chronic diseases is risking the sustainability of healthcare systems. In 2009, the World Health Assembly placed people and person-centredness at the centre of health care.^2^ Efforts to strengthen health systems and improve health outcomes should focus not only on technical and structural aspects but also the user experience and process of care by measuring features known to be essential for cost-effective personal health care.

The South African (SA) healthcare system has entered a period of major reform that includes primary healthcare (PHC) re-engineering and implementation of a national health insurance (NHI). The SA government has committed itself to improving PHC, the stated orientation of SA district health services.^3^ After 21 years of democratic governance, increased PHC funding and other measures to undo the legacies of apartheid policies and practices, there is widespread concern that desired health outcomes are not being achieved and that gross inequities in health status and access to services continue.^4,5^ In Western Cape (one of nine provinces), there are initiatives to improve the quality of primary care in line with the Provincial Department of Health’s Vision 2030. It includes a commitment to shift the focus from illness to wellness and from patient care to person-centred care and engaged leadership.^6^ This shift reflects significant changes in thinking and will require reorientation of staff, clinical practice, organisational management and resource allocation. Effect should also be given to the right of users to participate individually and collectively in the planning and implementation of their health care as espoused in 1978 in the Declaration of Alma-Ata, for example, through PHC stakeholder partnerships.^7^

Better health outcomes, reduced costs and reduced inequity are among the results of an integrated, publically funded health system that ensures user access to elements known to be essential for cost-effective PHC, and on which performance can be measured.^8,9,10,11^ The Primary Care Assessment Tool (PCAT) is a validated instrument^12^ used in developing and developed contexts internationally to determine the extent to which PHC is aligned with the evidence for cost-effective care based on users’ access to, and utilisation in their care, of these essential features (domains) – namely first contact access (to a primary care practitioner); ongoing care (relational continuity); comprehensive care; coordinated care; family- and community-orientated care and cultural competence. These are also known as the principles of family medicine which are embedded in primary care and family physician training in South Africa. Together with implementation of the NHI and provincial interventions, they provide SA with a significant opportunity to align PHC with evidence in favour of comprehensive, person-centred primary care. A baseline measure of PHC performance and organisation on these domains could help to guide reforms and other interventions prior to them taking root, and to monitor impact.

This study reflects a partnership between service providers (Western Cape Provincial District Health Services) and a tertiary education institution (Division of Family Medicine, University of Cape Town) to support efforts to improve primary care in the Western Cape Province. The main purpose of the study was to obtain a baseline measure of performance and organisation at comprehensive primary care facilities (PCFs) by determining users’ experience and managers’ and practitioners’ assessments of primary care. By surveying users, providers (practitioners) and managers, the study aimed to determine gaps between users’ experience of primary care and desired performance on the universally accepted essential primary care domains and to identify those that need strengthening and aligning with evidence-based care and with provincial and national health plans. Study objectives included determining the demographic profile of primary care users in urban and rural districts; measuring performance on 11 primary care domains and subdomains, and a total primary care score in selected health districts in Western Cape; describing user experiences of care by demographic variables; describing and comparing primary care user, practitioner and manager assessments (scores); comparing domain, subdomain and total primary care scores between districts; and reporting the main findings to PHC the user, practitioner and manager stakeholders.

In 2011, Cape Town had a population of approximately 3 810 000; 73.0% were uninsured and dependent upon the public sector, and the Cape Winelands district (CWD) had a population of approximately 768 300; 77.0% were uninsured and dependent on the public sector. The large uninsured proportion of the population skews the quadruple burden of disease towards the public sector, that is, infectious diseases (including child mortality and malnutrition), degenerative and chronic diseases, injuries and HIV-related diseases. Utilisation rates and annual patient visits at PCFs were 3.7/10 415 052 (Cape Town) and 3.4/1 978 282 (CWD).^13^

The investigators conducted the first South African PCAT study (unpublished) in two Cape Town metropolitan sub-districts (eight urban PCFs) in 2011. This second study was conducted in urban and rural PCFs – in the remaining six of the eight sub-districts in Cape Town (Southern = Western, Eastern = Khayelitsha and Northern = Tygerberg) and four of the five sub-districts in the CWD. All PCFs studied are comprehensive PHC facilities in the provincial health department.

Following the experience gained and lessons learned in the 2011 study in which the expanded (E) forms of the original USA PCAT questionnaires were used – adult expanded (AE), provider (practitioner) expanded (PE) and facility manager expanded (FE) – a cross-cultural adaptation and validation of the original expanded version was conducted and the expanded South African ZA PCAT produced for use in this study. The validation method, content and domain definitions of the ZA PCAT are described in an earlier paper.^14^ In summary, a combination of the face validation, Delphi and nominal group technique methods was used with two expert panels to achieve consensus on the relevance of PCAT domains and their items (domain questions) for use in SA. Consensus in favour of inclusion was achieved for all 9 domains. One new domain, the PHC team, was added. Of the original 95 items, 3 achieved < 70% agreement and were excluded; 19 new items were added; a few items needed rephrasing for local comprehension; the demographic section was adapted for local socio-economic conditions; and isiXhosa and Afrikaans translations of the ZA PCAT were developed.

## Method

This was a multilevel cross-sectional study of primary care users, practitioners and managers in six of eight urban and/or peri-urban and four of five rural sub-districts in Cape Town and CWD, respectively. The remaining urban sub-districts were studied in a pilot study in 2011. The 4 rural sub-districts were considered by CWD management as sufficiently representative of the whole district; including all five rural districts in the province was beyond the capacity of our study budget.

In each Cape Town substructure, PCFs were stratified into large, medium and small clusters as determined by user visits per month to ensure representation for size; opening hours (24 h and 8 h); and the 3 main languages spoken, that is, that demographic diversity was reflected across the metro sample. One PCF was selected from each stratum in collaboration with district managers. In the CWD, the largest PCF in each of the 4 sub-districts was included because they best reflected user and staff diversity, and the range of PHC services offered in CWD PCFs, that is, using cluster sampling, 13 PCFs covering 6 urban and/or peri-urban and 4 rural sub-districts were included in the sample. The outcome measures are performance scores on 11 key elements (subdomains) of primary care and total primary care score.

The user sample size calculation was based on primary care measures derived from a previous PCAT study (2011) with an estimated mean total primary care score between 2 PCFs of 2.5 and 2.9 with a standard deviation of 0.8. The minimum sample size required per PCF was 85 (α = 0.05 and a power = 90%). The total number of users that were interviewed in the 13 PCFs was 1432. The PCF with the smallest and the largest sample size was 97 and 123 users, respectively. All full-time doctors and nurse practitioners (140) working in the 13 PCFs and all PCF managers (87) working in the 10 sub-districts represented were invited to participate.

### Participant selection

For users, a systematic sampling method was used in each PCF. From the 2011 study, we estimated that each interviewer would conduct seven interviews per day. We aimed to survey approx. 20 users per day in each PCF so that approximately 100 interviews would be completed over 5 consecutive days from Monday to Friday. Using the average number of patients seen per day at each PCF (obtained from PCF records), the sampling interval (*n*th admission folder) was calculated by dividing the total number of daily admissions by 21 (3 interviewers x 7 interviews = 21). Counting from the top of the pile of admissions folders, every *n*th folder was assessed for eligibility using the inclusion and exclusion criteria. Only patients (users) ≥ 18 years of age with a minimum of three previous visits to their respective PCFs were included Responses to the ZA PCAT AE require patients’ experience of primary care over time; to be eligible respondents had to have visited the PCF at least three times prior to the day of the interview. Where a patient was not eligible or did not consent, the next folder was selected and so on. Fieldworker management and data quality control was by an experienced research assistant – a member of the fieldworker training team – who was on-site throughout the user data collection. Only full-time PCF practitioners and managers were included in the practitioner and manager samples; locums, interns and practitioners doing their community service were excluded.

The user survey was conducted by trained fieldworkers using the ZA PCAT AE. Fieldworkers selected for the study (CVs were scrutinised and interviews conducted) had a minimum of 12 years of schooling and previous research fieldwork training including data collection and research ethics. Each was fluent in at least two of the three official languages spoken in Western Cape – English, isiXhosa and Afrikaans. Following selection, the fieldworkers were trained to administer the ZA PCAT in a 3-day training workshop based on the original authors’ training manual.^15^ The manual content and training method were adapted and aligned with the cross-culturally validated ZA PCAT by the research investigator team. The practitioner ZA PCAT (PE) was self-administered – not necessarily on the same day – after it was explained by an investigator at respective PCF clinical practitioner meetings. In the metro, the manager ZA PCAT (FE) was administered by appointment by the trained study investigators. In order to avoid the cost of travelling to individual appointments in the rural sub-districts – a round trip of 240 km – rural PCF managers self-administered the ZA PCAT in sub-district manager groups after their monthly management meetings. An investigator explained the PCAT before completion and was available to respond to any queries.

Information meetings were held prior to the start of the study to brief and obtain cooperation of PCF staff, sub-district and district managers, and district directors. This included site visits to determine how best to adhere to the study protocol and yet keep disruption to clinic operations to a minimum. These meetings paved the way for meetings with stakeholders to report the main findings. Summary posters were placed in PCF waiting rooms and presentations made to user-represented PCF committees; hard copy reports and presentations were presented at PCF staff meetings (practitioners and managers) and sub-district and district manager meetings which included district directors. Verbal presentations and hard copy reports were also given to provincial health department senior management.

### Data analysis

The method of data scoring, analysis and formulation of results followed the steps in the PCAT manuals for the three expanded user, practitioner and manager PCAT versions (AE, PE and FE, respectively). These are obtained from Johns Hopkins Primary Care Policy Center.^15^ Data from each of the three informant groups were analysed separately. The PCAT Likert scale responses and analysis are the same for user, practitioner and manager questionnaires. Responses are scored on a 1–4 scale with 1 indicating ‘definitely not’, 2 indicating ‘probably not’, 3 indicating ’probably’, 4 indicating ‘definitely’. A fifth ‘not sure/don’t remember’ response option is scored as 2 (except for the comprehensive services domain where ‘not sure/don’t remember’ is scored as 0). The PCAT methodology calculates the score for each subdomain by summing the scores of the items in that subdomain (after reverse coding of items where required by the data analysis method) divided by the number of items to produce a mean score. Questionnaire data were entered into EpiData^16^ and exported to Stata version 12.0 for statistical analysis.^17^ The internal consistency of the scores for users was examined using Cronbach’s alpha coefficient. The Shapiro-Wilk test indicated that the PCAT scores were not normally distributed; hence, we constructed a binary variable. A score ≥ 3 is considered ‘acceptable to good performance’ and < 3 as ‘poor performance’. Multivariate binomial regression analysis^18^ was used to estimate the prevalence ratios (PRs) and assess the association between user’s primary care score and socio-demographic characteristics (independent variables). For all analyses, a *p*-value of less than 0.05 and a 95% confidence interval that did not span unity were considered the thresholds of statistical significance.

## Results

The ZA PCAT AE was administered to 1439 users (acceptance rate 90%). Only 7 user questionnaires (AE) were unsuitable for analysis due to missing or incomplete data; 1432 user questionnaires were analysed. Of the approximately 140 eligible practitioners in the PCFs studied, 100 completed the ZA PCAT PE (acceptance rate 71%). Of the 87 eligible PCF managers and deputy managers in the 10 sub-districts studied, 64 completed the ZA PCAT FE (acceptance rate 74%). All practitioner (100) and manager (64) questionnaires were complete and analysed.

Table 1 summarises the main user characteristics: 68.9% are female; 62.9% attended but only 9.9% completed high school and 2.7% had further education; 22.8% live in informal dwellings; and 35.3% are employed. The age distribution of users, 18–40 (34.8%), 40–54 (32.6%) and 55 + (32.6%), was similar in proportion. It also shows that 60.5% of the users perceived their health status as ‘good’.

**TABLE 1:** User characteristics.

Variables	Number	%
**Cape Town metro sub-districts**		
Southern-Western	351	24.5
Northern-Tygerberg	307	21.4
Eastern-Khayelitsha	339	23.7
Cape Winelands (Rural)	435	30.4
**Gender**†		
Male	443	30.9
Female	987	68.9
**Age-group**		
< 40	498	34.8
40–54	467	32.6
55+	467	32.6
**Health Status**†		
Poor	562	39.3
Good	866	60.5
**Employment**		
No	927	64.7
Yes	505	35.3
**Educational level**†		
No schooling or some primary schooling	525	36.7
Secondary (and higher)	901	62.9
**Type of dwelling**		
Informal	326	22.8
Formal	1,106	77.2
**Language**†		
English	244	17.0
Afrikaans	611	42.7
Xhosa	491	34.3
Other	83	5.8

† Missing values for the following variables in brackets: Gender (2); Health Status (4); Language (3); Educational level (6).

Table 2 shows the descriptive results of user scores by PCAT domain. The means reflect positive experiences in seven of the 11 primary care subdomains. The mean scores for first contact access, comprehensiveness (services provided), family centredness and community-orientated are below 3. Cronbach’s alpha estimates show acceptable reliability for 8 out of the 11 primary care elements (range 0.7–0.9). Coefficients for first contact utilisation (0.4) and coordination (information systems) (0.2) are far below the minimal acceptable score (Cronbach’s alpha = 0.7) to be reliable as a coherent domain.^12,19^ The Cronbach’s alpha coefficient for family centredness (0.6) is marginally below 0.7. Cronbach’s alpha levels were far below for first-contact utilisation and coordination information systems, indicating that the homogeneity of variances among items within the scale was low.

**TABLE 2:** Distribution of USER scores by PCAT subdomain (2013).

Subdomains	Number of items	Variable			Cronbach’s alpha

		Mean	SD	Range	
First contact – utilisation	3	3.1	0.6	1.0–4.0	0.4
First contact – access	17	2.5	0.4	1.3–3.6	0.7
Ongoing care	15	3.0	0.5	1.2–4.0	0.7
Coordination	10	3.2	0.7	1.4–4.0	0.8
Coordination (information systems)	3	3.2	0.7	1.0–4.0	0.2
Comprehensiveness (services available)	28	3.1	0.6	1.1–4.0	0.9
Comprehensiveness (services provided)	12	2.7	0.6	0.6–4.0	0.7
Family-centredness	3	2.8	1.0	1.0–4.0	0.6
Community orientation	6	2.3	0.8	1.0–4.0	0.8
Culturally competent	5	3.4	0.8	1.0–4.0	0.8
Primary healthcare team	7	3.4	0.6	1.3–4.0	0.7

PCAT, Primary Care Assessment Tool.

**FIGURE 1:**
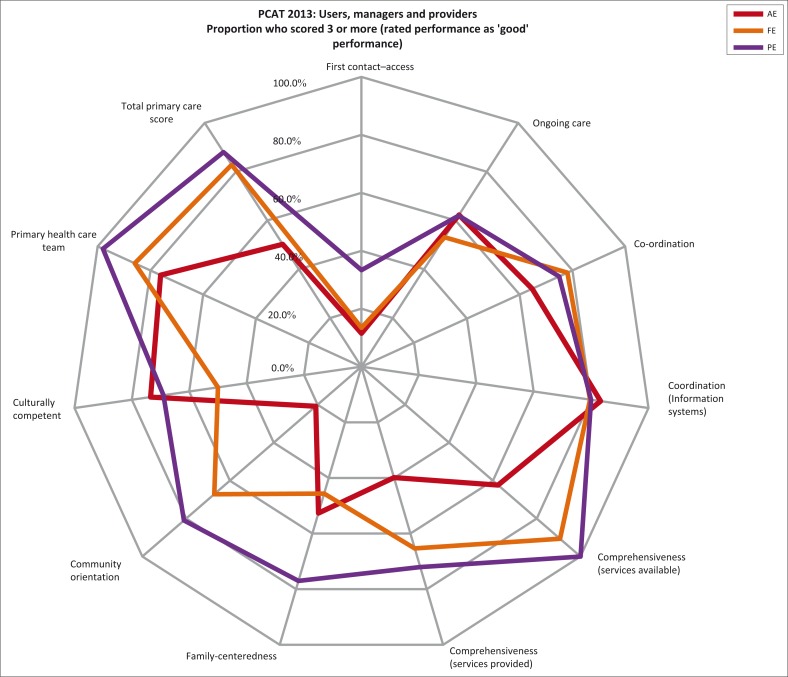
Comparison of acceptable to good performance scores for the 3 key stakeholders (i.e. scores ≥ 3).

Table 3 (graphically presented in Figure 1) shows the proportion of users, providers and managers who rated each domain as ‘acceptable to good’ performance. 11.5% of users scored performance on access as ‘acceptable to good’; 20.8% scored community orientation and 39.9% scored comprehensive services provided ‘acceptable to good’. The remaining subdomains were scored as acceptable to good by at least 50.0% of patients. Among the providers (doctor and clinical nurse practitioners), the lowest ‘acceptable to good’ score was for access (33.3%) and the highest for comprehensive services available (100.0%) and primary healthcare team (98.0%). Managers scored access (13.5%) and family centredness (45.6%) lowest; and comprehensive services available (90.6%) and primary healthcare team (85.9%) highest. The total primary care scores of provider and manager are similar but significantly higher than users’ total score.

**TABLE 3:** Proportion of users (AE), providers (PE) and managers (FE) who scored subdomains ≥ 3 (i.e. rated performance as‘good’).

Subdomains	AE (%)	FE (%)	PE (%)
		
	*n* = 1432	*n* = 64	*n* = 100
First contact – Access	11.5	13.5	33.3
Ongoing care	62.2	53.1	62.0
Coordination	64.8	78.1	75.0
Coordination (information systems)	83.3	79.7	80.0
Comprehensiveness (services available)	62.4	90.6	100.0
Comprehensiveness (services provided)	39.9	65.3	72.0
Family-centredness	52.7	45.6	77.0
Community orientation	20.8	67.2	81.0
Culturally competent	73.5	50.0	69.0
Primary healthcare team	76.1	85.9	98.0

**Total primary care score**	**50.2**	**82.8**	**88.0**

**FIGURE 2:**
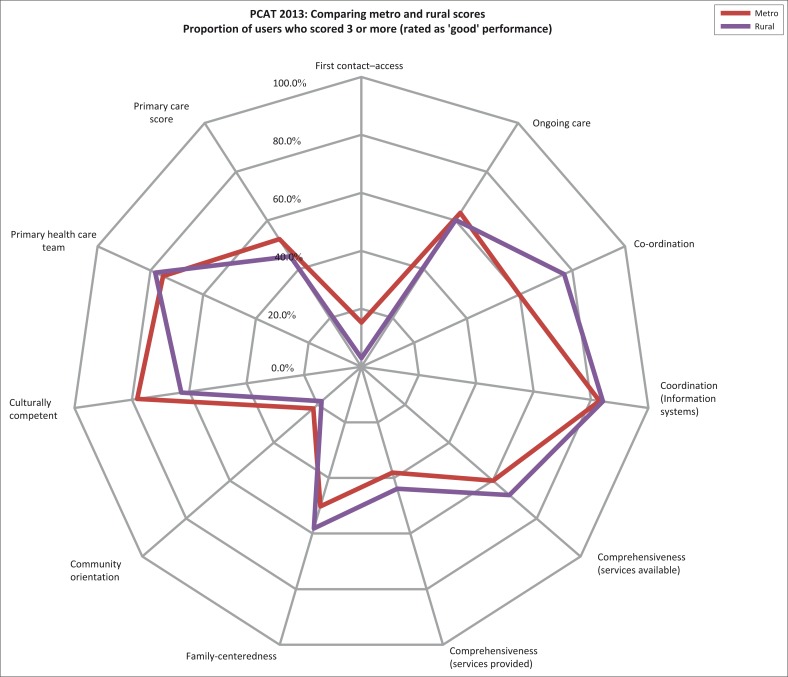
Comparing metro and rural user scores for acceptable to good performance (i.e. scores ≥ 3).

Table 4 (graphically presented in Figure 2) shows the proportion of users who rated each domain as ‘acceptable to good’ performance by urban and rural regions. Urban users scored first contact access and cultural competence significantly higher than rural users, whereas rural users scored coordination, comprehensiveness (services available), comprehensiveness (services provided) and family-centredness significantly higher.

**TABLE 4:** Proportion of users who scored subdomains ≥ 3 (i.e. rated performance as ‘good’) by metro and rural regions.

Subdomains	Metro (%)	Rural (%)	*p*-value
	
	*n* = 997	*n* = 435	
First contact – utilisation	90.8	92.4	0.312
First contact – access	15.2	3.0	< 0.001*
Ongoing care	63.1	60.2	0.305
Coordination	59.9	76.9	0.015*
Coordination (information systems)	82.9	84.1	0.579
Comprehensiveness (services available)	60.1	67.6	0.007*
Comprehensiveness (services provided)	38.1	43.9	0.039*
Family-centredness	50.3	58.2	0.006*
Community orientation	22.0	18.2	0.103
Culturally competent	78.1	62.8	< 0.001*
Primary healthcare team	75.2	78.2	0.231
Primary care score	52.4	45.3	0.014*

Multivariate analysis: The socio-demographic variables (Table 1) that were considered as possible predictors of user’s total primary care score were included in the binomial regression model as independent variables. The users’ total primary score, with a range of 1.7–3.7, was entered as binary dependent variable (a score ≥ 3 is considered ‘acceptable to good performance’. The results show that users who resided in Northern–Tygerberg and Eastern–Khayelitsha sub-districts; users ≥ 40 years; and users who rated their health status as good were significantly associated with a positive total primary care score (≥ 3 good):

$$\begin{array}{l} \text{N/T }PR=1.5[P=0.002\;CI:1.2-1.8];\text{E/K }PR=1.6[P<0.001\;CI:1.3-2.1];\\ \geq 40\text{ years }PR=1.2[P=0.017\;CI:1.0-1.5];\text{ Good health status}\\ PR=1.4[P<0.001\;CI:1.2-1.6] \end{array}$$ [Eqn 1]

Users were not surveyed for their responses to the waiting room posters. Only two functional PCF committees were identified for presentation of the main findings. They identified with the user findings; responses were positive and encouraging and showed a willingness to work with PCF staff to improve care. Manager and practitioner responses to reports are noted below.

## Discussion

The demographic findings (Table 1) show that public sector PHC facilities serve largely female users (68.9%); that more than a third (36.7%) of users have no formal schooling or only some primary school education; and that 23.0% live in informal housing in contrast to national figures (15.8% and 12.9%, respectively).^20^ Only 35.3% (female 34.3%; male 37.7%) reported any form of employment - whether part-time, full-time, informal or formal, that is, 64.7% were unemployed – by definition a larger proportion than ‘narrow’ unemployment defined as the proportion of unemployed actively seeking work – 24.3% nationally.^21^ Three socio-demographic factors (Table 1) emerged as predictors of total primary care score: users’ area of residence; age (≥ 40 years); and self-reported health status, that is, users who rated their health status as ‘good’. These were significantly associated with a positive total primary care score (≥ 3 = good). Although analysis of the demographic data suggests that sub-districts with higher unemployment and a lower education level are associated with higher total scores, it is not a conclusive finding in this study. A longer standing relationship with a PCF as the chosen provider may account for age as a predictor. Older users are more likely to have chosen a PCF over other options and therefore more likely to be satisfied. Users who rated their health as ‘good’ also rated primary care performance as better than those who rated their health as poor. A similar finding is reported in a Korean study where higher quality primary care was found to be associated with good self-rated health status.^22^ In a Brazilian study, users’ demographic and socio-economic factors were not predictors of primary care score.^23^ While socio-demographic factors such as unemployment, educational level and type of dwelling have not been shown to be predictors of primary care score in this study, they may nevertheless add to the complexity and challenge of providing primary care to communities where these are prevalent.^24^ Smith and Haggerty note that poor literacy is an independent health risk; high-literacy users may be less dependent on health service interventions and more able to act on providers’ advice.^25^

Most users (60.5%) perceived their health status to be good to excellent despite 75.8% reporting that they attended for a special medical problem; 56.0% had a physical, mental or emotional problem lasting or likely to last longer than 1 year. It is surprising that similar proportions who attended and who were not attending for a chronic condition rated their health as good (59.6% and 63.0%, respectively). It is unlikely that this self-rated ‘good’ health indicates good disease control in the majority of these patients; local chronic disease care audits generally reflect poor control. A recent study in 10 PCFs including facilities in our study confirms previous chronic disease audits – that the average proportion of users attending these facilities for chronic disease care is high (82.0%) and the quality of care and control for diabetes and hypertension is poor.^26^ The apparent discrepancy between perceived good health and evidence of poor disease control may be due to users’ interpretation of health and illness, that is, health is ‘good’ when not feeling ill or if function is not significantly limited. This may be particularly so in largely silent yet prevalent conditions such as hypertension and diabetes. The self-rated health status may be a function of a low expectation of heath care and disease control, that users were interviewed on site, and users’ association with respective PCFs. In the context of a quadruple disease burden, poor disease control, a 65.0% comorbidity^27^ and proportionally lower male user attendance, a falsely founded perception of good health has important implications for primary care practitioners, managers and initiatives aimed at improving the quality of care. Such a perception underscores the importance of good relational continuity necessary to build long-term therapeutic partnerships for effective management of chronic disease.

When comparing urban and rural districts’ user scores (Cape Town Metro and Cape Winelands, respectively), the patterns created by plotting the scores on the radar graph (Figure 1) are similar even though there are significant differences between their domain and total primary care scores (Table 4). The similar patterns suggest similar strengths and weaknesses in performance on the essential features measured – expected given standard provincial PHC packages and treatment guidelines, management training and protocols, and comparable clinician training and practice. However when comparing user, provider and manager scores (Table 3; Figure 1), the patterns are different; differences between the three key stakeholders are greater than the differences when comparing user scores by region (not shown). Users scored comprehensive services available, comprehensive services provided and community orientation, significantly lower than providers and managers. In general, the providers (doctors and CNPs) scored PHC performance on most domains higher than both managers and users.

Managers’ scores are closer to those of users’ experience (Table 3). Practitioners’ scores are optimistic relative to managers and users – a cause for concern, practitioners being the frontline providers of care. It is hoped that these findings will increase awareness among managers and practitioners of the gaps between the user experience and perceived performance; and encourage a search for ways to reduce the gaps between current and desired performance, for example, by developing and implementing interventions aimed at improving staff adherence to evidence-based care. The validity of comparing user (AE), practitioner (PE) and manager (FE) domain scores may be debatable given their different roles and perspectives. However, a co-author of the original PCAT agrees^1^ they can be compared.^28^ We can only speculate on reasons for the optimistic practitioner scores relative to users and managers. Much of what the PCAT measures are process transactions which take place during user consultations with practitioners - the heart of personal primary care. The relatively optimistic practitioner scores may reflect concern at being judged by low scores. Viewed together, practitioner and manager scores for total primary care are optimistic relative to users’ scores, that is, users’ experience of primary care is significantly different to what managers and providers think they are delivering. Further research is required to explain this; the findings could help to identify and implement interventions to improve practitioner and manager performance on essential domains of primary care. Joint consideration of the disparate performance scores by these key stakeholders can serve as an opportunity to build a primary care stakeholder partnership that includes generating, implementing and monitoring appropriate interventions aimed at improving performance thereby improving health outcomes.

*Access* involves the extent to which primary care services are accessible to users when needed. Domain items include opening times, waiting times and staff attitudes, that is, both structural and process factors. Relative to other scores, performance on first contact access was rated as poor by users, practitioners and managers (11.5%, 33.5% and 13.5%, respectively) suggesting agreement that access needs attention. Of note is that when measured on a scale of 1–4, a Canadian study reported a mean score of 2.21 (3 being acceptable) for first-contact access^29^ – a finding similar to our 2.5 (Table 3). Stakeholder agreement on poor access contrasts with the high user score for first contact (utilisation), that is, high user affiliation with PCFs in urban (90.8%) and rural (92.4%) regions (Table 4) – a finding supported by a high proportion of users reporting an association with their respective primary care facilities of > 5 years. High user utilisation and affiliation contrasts with the lower user score (62.2%) for ongoing care (Table 3) and suggests missed opportunities to strengthen relational continuity and to build strong therapeutic user-practitioner and user-facility relationships by practicing good continuity of care. This is an important finding given the influence of continuity on other domain performance (discussed below).

*Ongoing (continuing) care* refers to the use of a regular source of care over time that is not limited to certain types of healthcare needs, that is, health care is provided regardless of the presence or absence of disease. It includes building a user–practitioner relationship based on trust and practitioner knowledge of patients and their families. Although 62.2% of the users rated ongoing care overall as good (as did the practitioners), it is generally accepted among managers and practitioners in the districts studied (confirmed at our report-back meetings with practitioners and managers) that relational continuity of care is poor and that the service is not structured to encourage nor support relational continuity. The higher than expected user score for ongoing care in this study could be due to a low user expectation. Here too, practitioner scores are relatively optimistic regarding how well and how much they know about their patients. Sub-analysis of the 15 items in this domain may help to explain these discrepancies. Detailed analysis of the ongoing care domain was not an objective of this study; however, it deserves more attention here. Relational continuity is considered the most important element (principle) of primary care.^30^ It is embedded in this 15-item domain (e.g. item D1: *Do you see the same practitioner at each primary care visit?* No = 70.0%). Two unpublished audits of continuity conducted in Cape Town sub-districts (also included in this study) reported poor continuity of care. When continuity was defined as seeing the same practitioner for at least 2/3 (66.0%) of the consultations, it was present in 21.4% (95% CI: 13.4–31.3), whereas 92% (95% CI: 84.6–96.8) of the respondents preferred seeing the same doctor at each consultation.^31^ A large body of evidence summarised in a review by Haggerty et al.^32^ shows that poor continuity results in fragmented care and poor and costly outcomes. The authors note that policy reports and charters call for enhancement of continuity in healthcare delivery, for example, the Ljubljana Charter on Reforming Health Care urges that heath reforms reinforce continuity.^33^

It is difficult to envisage other components of ongoing care receiving the necessary attention in the absence of continuity – such as users being known and understood by their practitioners. Involvement of consecutive practitioners in an individual’s primary care leads to reduced accountability – labelled the ‘collusion of anonymity’ by Balint.^34^ Such practice is likely to be aggravated where there is a high patient-to-practitioner ratio, a feature of the public sector where practitioners generally see large numbers of patients per day. Under such circumstances comprehensive care also suffers. A study examining the quality of chronic disease care found that models of primary care with more than four family physicians and high patient ratios performed less well on PCAT domains than those with fewer family physicians and lower patient–practitioner ratios; and that community health centres in Canada and the USA performed better than office-based family physicians and hospital outpatient clinics.^35^ In a systematic review examining the relationship between (sustained) continuity of care and the quality of care, continuity was found to be associated with patient satisfaction, decreased hospitalisations and emergency department visits, and better acceptance of preventive services particularly in the care of chronic conditions.^36^ Continuity, when practiced by primary care professionals in a regulated health system is associated with better health outcomes and lower costs than in fragmented, unregulated systems – such as market driven systems where patients initiate visits to medical specialists.^8^ It is worth noting that continuity is increasingly being highlighted in SA national and provincial health policy documents such as the Western Cape’s Vision 2030.^37^ Primary care practice should be aligned with such policy initiatives.

*Coordination* (subdomains: coordination of information systems and coordination of information) links healthcare events and services, and requires mechanisms to communicate and incorporate information into patient care plans. It includes the responsibility and obligation to transfer information to and receive it from other resources involved in the patient’s care (coordination of information systems), and to develop and implement an appropriate plan for healthcare management and disease prevention. All three stakeholders scored performance on coordination of information as good – both rural and urban – reflecting longstanding public sector record keeping and referral practice. Access to secondary and tertiary care (gatekeeping) is by referral only from a PCF practitioner. There is usually exchange of at least some patient information between PCFs and referral hospitals using paper-based or electronic referral letters. While misplaced or misfiled user records are not uncommon and fragments care, records are generally available at visits. User access to their records is also good; in many PCFs patients carry their own records between service points, for example, from the practitioner to pharmacist. In contrast, performance on the sharing of test results with users shows a significant difference between users on the one hand and managers and practitioners on the other. Relative to users’ experience, managers and practitioners are optimistic about their performance. The lower user score may indicate that results, for example, of special investigations are not communicated in ways they understand. This needs further research. Effective information sharing is an essential ingredient for a therapeutic user–provider partnership. We are unable to explain the difference between the urban and rural scores.

*Comprehensive care* (subdomains: comprehensive services available and comprehensive services provided) provides a range of essential personal health services that promote and preserve health as well as services for illness and disability, and arranges access to services elsewhere for uncommon or special needs. The importance and impact of comprehensive care is unique to primary care compared with other clinical disciplines. Comprehensive care provided includes the opportunistic provision of information and screening for health promotion, disease prevention and early detection guided by epidemiology and the health profile of user communities. Users, practitioners and managers scored comprehensive care provided lower than comprehensive services available. User scores (39.9% and 62.4%, respectively) were considerably lower than practitioner and manager scores. The scores suggest that while services are available at PCFs, they are not applied at an acceptable level of performance; and that practitioners and managers are optimistic about their performance. A study involving 14 PCFs in Cape Town (including PCFs in this study) reported a missed opportunity rate of 25.0% – 46.0% (depending on a strict or loose definition) for reproductive and mental health^38^ – a finding supported by more recent studies showing poor chronic disease control and staff adherence to policy.^26,39^ A Quebec study involving 100 PCFs across urban and rural settings found that even among users who had regular family physicians and reported experiencing high relational continuity, only 56.0% reported having age- and sex-appropriate health promotion and preventive issues addressed; 38.0% of those without family physicians (e.g. those attending walk-in clinics instead) had these addressed.^29^ The relationship between comprehensiveness and continuity was noted above. While relational continuity does not guarantee good performance on comprehensive care (a narrow, disease-oriented approach is still possible), the evidence that continuity is independently associated with improved outcomes^32^ suggests that comprehensive care is a function of continuity and likely to improve with improved continuity.

*Community orientation* recognises the primary care needs of a defined (practice) population. The effective delivery of services to individuals and communities is based on an understanding of community needs and the integration of a population perspective in the provision of primary care. Primary care providers contribute to and participate in community assessment, health surveillance, monitoring and evaluation. When reporting our findings to stakeholders, PCF managers were surprised by the low user score for community orientation (20.8%) and felt that the community may not be aware of community-based services (CBS) such as those rendered by a third party on behalf of the provincial health department. The score gap between users and managers and providers for community orientation was a consistent finding across all sub-districts and PCFs – urban and rural). This is an important finding given that community-orientated primary care is a key feature of PHC and the SA Health Department’s PHC re-engineering initiative. This may in part be due to absent or inadequate community involvement and messaging about existing CBS, that is, the low user score may indicate inadequate communication of services offered to users and user bodies. While there has been a significant shift to CBS, elements of a disease orientation nevertheless remain (e.g. TB and HIV home-based care and chronic disease adherence support groups) in contrast to the comprehensive PHC approach described in reviews of former local CHW programmes^40,41^ and being practised in recently established ward-based out-reach teams (WBOTs) in wards in Tswane and Soweto – projects of the Universities of Pretoria and Witwatersrand, respectively, and the Gauteng Health Department. The Brazilian comprehensive healthcare team-based family health programme (on which the WBOT is modelled) is associated with a higher total primary care score as well as higher scores for comprehensiveness, family and community orientation when compared with the traditional health system.^23^

*Family-centred care* considers the impact of the family on the genesis and prevention of ill health, as well as the response to both medical and psycho-social interventions. It recognises and incorporates knowledge of the family context (e.g. resources, risk factors and social factors) into the planning and provision of primary care. As noted above, practitioner scores are optimistic (77%) relative to users and managers (52% and 45%, respectively). ‘Thinking family’ is the distinguishing feature of family-centred primary care. Family-centred thinking is an approach to routine primary care of individuals that considers the family or household as an integral part of information gathering, clinical reasoning and patient care.^42^ There is considerable evidence to show that family-centred care improves health outcomes.^43^ Narrowing the gap between user experience and provider-rated performance can therefore be expected to yield better outcomes.

*Cultural competence* incorporates cultural references into the provision of primary care. Culturally competent services are acceptable to people in the community who may be distinguished by common values, language, heritage and beliefs about health and disease. It implies that their views are determined and incorporated into decision-involving policies, priorities and plans related to the delivery of healthcare services. Managers’ performance scores on cultural competence are considerably lower (50.0%) than users and practitioners (73.5% and 69.0%, respectively). The user score may reflect staff demographics such as language and ethnicity increasingly approximating those of the users at many PCFs. The lower manager score could be due to items included in the manager PCAT that determine whether specific interventions to address diversity and transformation are part of continuing staff development, suggesting managers would like more attention given to these. The oversight and governance roles of managers would include having to deal with problematical interactions between staff and users; they may therefore be more aware of service shortcomings. Cultural competency is a complex and dynamic concept.^44,45^ The PCAT definition which determines the items in this domain, for example, language competency and sensitivity to traditional heath beliefs and practices, may be limited relative to the complexities in SA society that have to be negotiated daily and driven by historical, sociopolitical, ethnic diversity and other factors.

*PHC team* (composition) determines which members of the team (other than practitioners) are present, that is, whether users have on-site or nearby access to these for at least some days of the week. Managers (score 85.9%), given their leadership and management roles, are best placed to provide information on PHC team composition at their respective PCFs. Users (score 76.1%) who do not need the services of team members other than nurses and doctors may be less aware of them. The lower user score may also reflect less than satisfactory inclusion of other team members by doctors and CNPs – supporting the contention above that the opportunities to use the services available in a comprehensive approach to care are frequently missed. This notwithstanding, the PHC team domain may be of limited use in the ZA PCAT; the results merely indicate that on the whole, core human resources are available at PCFs. Such information can easily be obtained from managers and PCF records. Assessing PHC team functioning and effectiveness would be more useful in such an audit; its absence is a shortcoming of the tool. (A validated 7-item instrument^46^ that assesses team effectiveness^46^ since being included in the provider (PE) and manager (FE) ZA PCAT is being tested.)

At the report-back sessions involving district managers we observed a desire to understand the user experience in more detail in order to identify ways to improve PHC, suggesting that appropriate interventions are likely to be well received. This is especially important for chronic disease care given its contribution to the growing disease burden and poor outcomes despite the high costs of care in local PCFs.^26,27^ It augurs well for other important initiatives, for example, to improve comprehensive care by improving health promotion and disease prevention, and reducing missed opportunities in primary care.

### Study limitations

Our facility-based sample excluded non-users of PCFs, including those in poor health too unwell to attend. Among the non-user group there is likely to be a subgroup of former users who for one or more reasons – including service dissatisfaction – no longer attend but have important information on the user experience. We chose a facility-based sample to identify actual PCF users in order to audit public sector primary care especially given the considerable effort and resources committed to improve public sector care – such as the National Department of Health’s PHC re-engineering initiative.^47^ A community survey would have required a much larger sample and budget to achieve the calculated user sample size for the facilities studied. Sampling users on-site also made a rapid appraisal of current primary care performance, possible and affordable in a resource-constrained setting. Facility-based sampling was used in a Brazilian PCAT study for similar reasons.^23^ The PCF-based sample is likely to result in a selection bias towards positive scores and better health status.

Sampling each PCF over 1 working week (5 days) may not represent the user experience during other weeks of the year given changing operational and seasonal effects. However, user responses were determined by the past experience of primary care which would have reduced the impact of operational and seasonal effects and therefore on the results.

Users’ responses depend on their knowledge of the service and recall of past experience; an element of recall bias is therefore expected. This may have been tempered by respondents’ length of association with their clinic (94.0% for 3 years or more; 59.6% for 5 years or more).

On-site surveys are known to be less reliable; respondents are less likely to report negative views when their perceptions and experiences of a service are **e**licited on-site in face-to-face interviews.^48,49^ Measures to reduce the impact included assuring respondents that the survey was anonymous; that identifiable and personal information was not required; that their responses would not affect usual care; and that the survey aimed to improve services based on users’ collective experience of care. Healthcare users are encouraged to report poor health service delivery by using complaints boxes in health facilities and a dedicated telephone line. Although the PCAT is not a satisfaction survey, 10.0% of the respondents spontaneously offered details of negative experiences and/or ideas to improve the service – recorded on a blank page by the interviewers – suggesting they were comfortable responding to the questions. Haggerty et al., in a review of instruments assessing primary care performance, note that users are less likely to assess performance negatively if they are unable to pinpoint the cause of their negative experiences. Assessments therefore tend to be skewed towards the adequate to excellent range; negative assessments are more likely to be true negatives, that is, have a higher specificity than positive assessments. The authors suggest that it may be more accurate to report the ‘percentage of less-than-positive scores’ to avoid positives scores ‘masking’ negative scores.^50^

As noted above, self-reported ‘health status’ is a subjective assessment. Differences between perceived health status and the audit findings may reflect a limitation of the PCAT. However, while health status can be expected to influence users’ experience and therefore users’ scores, this does not diminish their experience and the validity of their scores.

Differences between user, provider and manager scores may be due to different interpretations of subdomain items (questions) – a concern raised by managers and staff during report-back sessions. While item content and phrasing for managers and practitioners are necessarily determined by their respective roles, subdomain definitions (Appendix 1) are same for users, managers and practitioners. Any limitation notwithstanding, the significant difference in performance scores between users on the one hand and practitioners and managers on the other – for example, for community-orientated primary care especially following increased emphasis on CBS for chronic disease care – is concerning. Optimistic scores may lead to a false sense of doing well when in fact improvement is needed. It is noteworthy that during our report-back sessions, district managers generally accepted users’ scores as reflecting their (users’) experience of primary care and low scores as indicative of subdomains needing attention.

Caution is needed when applying the study results to other PCFs and districts in the province. Although sub-district managers had a say in selecting PCFs in the Cape Town metro, it was within strata and where options were comparable. The districts sampled are the responsibility of one provincial health authority; PCFs operate in similar contexts with similar staff training, operational plans and constraints. Similarly, caution should be exercised before generalising to other provinces given the resource disparities between provinces.

## Conclusion

These are the first PCAT audit results using the cross-culturally adapted ZA PCAT (expanded form) and the first PCAT study in Africa. Primary care elements shown universally to be essential for cost-effective primary care were audited; elements that need strengthening – especially with respect to users’ experience - were identified. The results also suggest a need to reduce, or at least explain, gaps between the users’ experience of primary care and practitioners’ and managers’ assessment of the care they provide. They highlight a need for better alignment with international best practice as well national and provincial health policies that increasingly promote the importance of the user experience of care in health sector reform. The findings were acknowledged by PCF managers – and district-level managers present – at the report-back sessions; managers did not find users’ account of their experience surprising. While caution should be exercised before generalising the study findings, the results are in keeping with much of what is already known about primary care in South Africa – as noted in the introduction to this paper. We believe the results provide an important baseline measure of urban and rural PHC performance and organisation in the Western Cape Province – needed to determine the impact of imminent national and current local reforms. Notwithstanding the study limitations, the results have potential to guide the implementation of reforms.

## Recommendations

An audit is of little use if the information generated is not used to identify and guide interventions – in this case directed at strengthening PHC. Rather than generating a list of recommendations covering the range of domains, we mention two recommendations: building PHC stakeholder partnerships and user registration with a PCF – which we consider necessary for more specific interventions to succeed.

Finding common ground among stakeholders by discussing and interpreting results together could form the basis of strong PHC stakeholder partnerships in the communities served to strengthen efforts to achieve health sector reform goals embodied in National Health Department initiatives – such as PHC re-engineering and the ‘ideal clinic’ – and the Western Cape Provincial 2030 Plan. In keeping PHC and the Alma-Ata Declaration, communities should be recognised as key stakeholders in partnerships that generate, prioritise, select, implement and monitor interventions – for example, using the quality improvement cycle in a participatory action approach and the ZA PCAT to re-audit performance.

User registration with a local PCF is not a feature of public sector primary care in South Africa where users are free to use PCFs wherever they wish. One-third of public sector users regularly seek primary care from more than one provider including the private sector.^31^ Limited access to 8-h facilities and long waiting times are among the contributing factors. However, user acceptance of registration with one PCF – especially among the indigent who have few choices – is likely to be a challenge. South Africans were forced by law to live and use services in designated areas prior to 1994. Uptake will need strong user–provider partnerships, trust and improved services. The high user–facility association (noted under the *access* domain above) is encouraging and suggests a likelihood of some success if user registration is properly encouraged.

The PCAT subdomains are synonymous with the well-known principles of family practice; the findings therefore suggest an important role in reform for primary care (family) physicians specifically trained to practise continuing, comprehensive, person-centred, family and community-orientated primary care in a range of contexts. These results should be considered when reviewing primary care practitioner and manager training. They should also be considered in PHC policy formulation and the research agenda.

## Footnotes

1 Shi, L. ‘It is OK to compare patients’ perceptions of experience with those of providers and managers provided the sample size is adequate’. E-mail to Graham.Bresick@uct.ac.za 8th January 2012.
